# Efficacy and safety of intense pulsed light compared to diode Laser for hair removal: a randomized controlled trial

**DOI:** 10.1007/s10103-026-04904-6

**Published:** 2026-06-06

**Authors:** Renata Taylor de Brito Barros, Olívia Harumi Araújo Sato Stella, Thais Rodrigues Galache, Luciana Camargo Khachikian, Richard Eloin Liebano, Christiane Pavani

**Affiliations:** 1 Biophotonics-Medicine Postgraduate Program, Universidade Nove de Julho, São Paulo, Brazil; 2 Universidade São Francisco, Bragança Paulista, Brazil; 3 Department of Rehabilitation Sciences, University of Hartford, West Hartford, USA

**Keywords:** Hair removal, Selective photothermolysis, Intense pulsed light, Diode laser, Randomized clinical trial

## Abstract

To compare the efficacy and safety of intense pulsed light (IPL) versus diode laser (DL) for female axillary hair removal in a randomized split-body trial. Women with Fitzpatrick skin types I–IV received IPL (690-nm filter) and DL (810 nm) on contralateral axillae with randomized side assignment. Four monthly sessions were performed. The primary outcome was hair count in a standardized 4-cm² area. Secondary outcomes included hair shaft thickness, pain intensity (numeric rating scale), participant satisfaction, evaluator-rated improvement (modified GAIS), quality of life (WHOQOL-bref), and adverse events. Participants and outcome assessors were blinded to side allocation. Forty-eight participants initiated treatment; 36 completed all sessions and 28 attended the 4-week post-treatment assessment (S4). Hair counts and hair shaft thickness decreased significantly over time with both modalities. At S4 (primary endpoint), the intention-to-treat analysis showed no statistically significant between-modality difference in hair counts; at 30-week (around 7 months) follow-up, DL showed more favorable objective outcomes (lower hair counts and thinner residual shafts). Pain scores were consistently higher with DL. Adverse-event profiles differed: erythema was more frequent with IPL, whereas perifollicular edema and carbonization were more frequent with DL; events were transient and required no medical intervention. Exploratory stratification by Fitzpatrick skin type showed no consistent pattern of increased adverse events in higher skin types. Satisfaction favored DL, while WHOQOL-bref scores did not change significantly. IPL and DL were both safe and effective for axillary hair removal in women with Fitzpatrick skin types I–IV. DL yielded more favorable longer-term objective outcomes but greater discomfort and transient procedure-related adverse effects. Trial registration: NCT06179186, 23 December 2023.

**Supplementary Information:**

The online version contains supplementary material available at 10.1007/s10103-026-04904-6.

## Introduction

Long-term hair removal is widely sought for both aesthetic and clinical reasons, particularly in populations living in warmer climates, such as those in Latin America, where cultural and lifestyle factors contribute to a high demand for durable hair reduction [1, 2]. Although conditions such as hirsutism and hypertrichosis may negatively impact quality of life [3, 4], the demand for hair removal extends beyond medical indications, reflecting broader sociocultural preferences in otherwise healthy individuals.

Light-based technologies have become central to clinical practice for hair reduction, with multiple devices available, including alexandrite, diode, and Nd: YAG lasers, as well as intense pulsed light (IPL) systems [5–8].

Among these, IPL is widely used due to its relatively lower cost, versatility, and broad range of dermatological applications, including treatment of vascular lesions (e.g., rosacea and port-wine stains), acne, and photorejuvenation, in addition to hair removal [9–15]. This versatility contributes to its widespread adoption in clinical and aesthetic settings.

In contrast, diode laser (DL) systems emit coherent, near-infrared light (≈ 810 nm), allowing targeted follicular heating and potentially more efficient energy delivery. Although DL is often considered a more advanced technology for hair removal, it is generally associated with higher costs and less versatility compared to IPL. In this context, comparing the effectiveness of IPL with DL is clinically relevant, particularly in real-world settings where treatment choice is influenced by both economic and practical considerations.

Despite widespread use, randomized trials comparing IPL and DL under standardized conditions remain limited. Available studies generally show significant hair reduction with both modalities, but substantial heterogeneity in design (parallel vs. split-body), treatment parameters, anatomical sites, follow-up duration, and outcome assessment [16–21]. While some studies suggest greater hair reduction with DL, others report comparable outcomes between modalities, or, in some cases, favorable outcomes with IPL. Moreover, many trials have presented modest sample sizes, been conducted predominantly in lower Fitzpatrick skin types or narrowly defined clinical groups, limiting the generalizability of their findings to mixed-skin-tone populations, such as those commonly observed in Latin America.

Taken together, these limitations indicate that the comparative performance of IPL and DL remains incompletely understood and that further studies using intraindividual randomized designs, standardized treatment parameters, and longitudinal assessment are needed to better characterize treatment response and durability. Therefore, the aim of this randomized split-body trial was to compare DL (810 nm) and IPL (690 nm filter) for axillary hair removal in women with Fitzpatrick skin types I–IV under standardized treatment conditions. By incorporating repeated assessments across treatment sessions and follow-up, as well as both objective and patient-centered outcomes, this study seeks to provide a more detailed and clinically relevant evaluation of treatment efficacy, tolerability, and durability in a representative Latin American population.

## Methods and analysis

### Trial design

This was a randomized, double-blinded (participant and outcome assessor), within-person (split-body) controlled trial, in which each participant received both interventions with randomized side allocation (1:1 ratio; DL in one axilla and IPL in the contralateral side). The protocol was approved by Research Ethics Committee of Universidade Nove de Julho (UNINOVE, approval no. 6.516.657) and registered at ClinicalTrials.gov. All participants provided written informed consent before enrollment. The CONSORT checklist was used when writing this manuscript [22].

### Participants

Eligible participants were women aged 18 or older with axillary hair and Fitzpatrick skin types I–IV. Exclusion criteria: use of systemic medications interfering with hair growth (e.g., photosensitizing agents, isotretinoin, tetracyclines, anticoagulants), pregnancy or lactation, history of vitiligo, epilepsy, psoriasis, malignancy, immunosuppression, or active herpes simplex infection. Women with sun-induced inflammatory sensitivity, hypertrophic or atrophic scars in the axillary region, tattoos in the treatment area, prior laser hair removal, or recent (< 30 days) hair removal by waxing or mechanical epilation were excluded. Postmenopausal women or those with prior oophorectomy were not eligible. Recruitment started in December 2023, and the study ended in November 2024. It was conducted at Universidade Nove de Julho, São Paulo, Brazil.

#### Sample size

The sample size calculation was based on results from a previous study comparing hair count after using DL and IPL under similar parameters [16]. The between-group difference was 3 (37 minus 34) and the effect size calculation was performed considering a standard deviation twice the magnitude of the between-group difference. The sample size was determined using G-Power software (Universitat Kiel, Germany) assuming a t test to measure difference between two dependent means, type I error 5%, power of 95%, and a 15% expected dropout rate, reaching a minimum of 62 participants to be included in the study. Recruitment was discontinued due to feasibility constraints; a post hoc power assessment based on the observed effect size indicated > 80% power.

#### Randomization

Randomization was performed at the side level (contralateral axillae, split-body design). An independent researcher generated the allocation sequence using block randomization without stratification. Participants were assigned to one of two side-allocation patterns: (1) IPL to the right axilla and DL to the left axilla, or (2) IPL to the left axilla and DL to the right axilla. Allocation concealment was ensured using sequentially numbered, opaque, sealed envelopes prepared by an independent researcher. After baseline assessment, the treating investigator opened the next envelope to reveal the side assignment and delivered both modalities accordingly.

This within-person (split-body) design was selected to minimize interindividual variability, as different body sites within the same individual are expected to respond in a comparable manner to treatment. Randomization of treatment side (left vs. right) was performed to avoid systematic bias related to laterality and to ensure balanced distribution of any baseline asymmetry between interventions.

### Intervention

At baseline, both axillae were cleansed with 0.5% alcoholic chlorhexidine and shaved with disposable razors. Assessments and standardized photographs were obtained before treatment, followed by application of a water-based neutral gel. Treatments were delivered with IPL (Light Pulse, HTM Eletrônica, Brazil) and diode laser (LightSheer ET/ST, Lumenis, Israel). Parameters were standardized across modalities, and participants underwent four treatment sessions performed at approximately monthly intervals (26–31 days, Table 1). Spot size differed between devices (3.96 cm² for IPL and 0.81 cm² for DL), reflecting inherent characteristics of each system. The DL handpiece provided integrated contact cooling for the tip, which was maintained continuously during treatment, as no direct temperature adjustment is available. The IPL device was operated using the maximum available contact cooling setting for the tip throughout all sessions to enhance epidermal protection and patient comfort. Applications were performed in spot mode with full coverage of the axillary area. An aloe vera–based soothing gel was applied after each session. All procedures were performed by the same trained operator.

**Table 1:** Treatment parameters

Parameter	IPL	DL
Central Wavelength [nm]	> 690	800
Spectral Bandwidth [nm]	510	10
Operation Mode	Quasi-continuous	Quasi-continuous
Peak Power [W]	6600	1600
Beam Area at Source/Target (SPOT SIZE) [cm^2^]	3.96	0.81
Irradiance at Target [W/cm^2^]	1667	1975
Pulse Duration [ms]	10	10
Fluence [J/cm^2^]	25	25
Radiant Energy per Pulse [J]	99	20.25
Anatomical Site of Application	axilla	axilla
Application Technique	Spot/Contact	Spot/Contact
Number of Sessions	4	4
Interval Between Sessions [days]	26–31	26–31

Photographic documentation, dermoscopic imaging, hair counts, and outcome assessments were performed by an independent blinded evaluator. The operator was not blinded; participants, outcome assessor, and data analyst were blinded to allocation. Participant blinding was ensured using opaque blindfolds and protective goggles; devices were activated before participants entered the room to minimize auditory cues.

Participants were instructed to avoid additional shaving or hair-removal methods in the axillary area during the study. Adherence was reinforced via WhatsApp reminders sent one week and one day before each visit.

### Outcome measurements

The primary outcome was hair count measured within a standardized 4-cm² area at the central axilla, assessed at baseline (S0), before each session (S1–S3), and 4 weeks after the last session (S4; prespecified primary endpoint). An additional follow-up (FU) assessment was performed 30 weeks after treatment (around 7 months), which allowed assessment of sustained effects. Secondary outcomes were hair shaft thickness, pain intensity during treatment, evaluator-rated aesthetic improvement (hair-density reduction), participant satisfaction, quality of life, and adverse events.

**Fig. 1:**
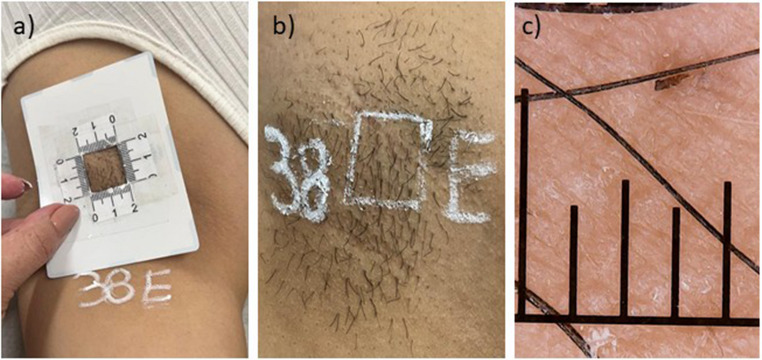
Photograph standardization. (**a**) positioning of the 75 cm² demarcation guide with a central 4 cm² region with the participant identification number and indication of hemibody (D indicating right and E indicating left); (**b**) Photographic record with the demarcated 4 cm² area; (**c**) Image of the hair captured using a dermatoscope for hair thickness measurement. The inserted ruler has 1 mm subdivisions

For hair counting, the axilla was labeled with participant ID and side (Fig. 1). A 75-cm² template with a central 4-cm² window was aligned to the arm axis to ensure reproducible positioning, as previously described [23]. The borders were marked with a white skin-marking pencil, and photographs were captured using an iPhone 11 under ambient lighting. Hair counts were performed by a blinded assessor on magnified images using ImageJ (NIH) with manual counting of all emerging hairs within the 4-cm² window (including vellus hairs) [24]. Hair counts were obtained within a standardized 4 cm² assessment area, nonetheless, for reporting purposes, values were converted to hairs/cm² to facilitate comparison across studies.

Hair shaft thickness was assessed from dermoscopic images acquired at each time point using a contact dermatoscope (5× magnification) with a metric scale (1-mm subdivisions). Images were calibrated in ImageJ, and three randomly selected intact shafts within the assessment area were measured; mean values were used for analysis.

Pain intensity was assessed verbally during each session using an 11-point Numeric Rating Scale (NRS; 0 = no pain, 10 = worst imaginable pain) [25–27]. Although a 10-cm visual analogue scale (VAS) was prespecified, a verbal NRS was used because protective eyewear made visual marking impractical and would have disrupted workflow.

Aesthetic improvement (hair-density reduction relative to baseline) was rated by a single independent evaluator blinded to allocation using a modified Global Aesthetic Improvement Scale (GAIS) based on paired standardized photographs: 0 = no improvement (0%), 1 = poor (> 0–25%), 2 = fair (25–50%), 3 = good (50–75%), and 4 = excellent (75–100%) [28].

Participant-reported outcomes at follow-up were assessed using a single-item measures with the aim of capturing specific aspects of the treatment experience. These included the perceived discomfort during sessions (5-point ordinal scale), the number of sessions associated with discomfort (0–4), the overall satisfaction with results (5-point ordinal Likert-type scale) [29], and the perceived overall improvement (0%, 25%, 50%, 75%, or 100%). Each item was analyzed independently and was not intended to represent a composite or validated multidimensional instrument.

Quality of life was measured at S0 and FU using the WHOQOL-bref (26 items; four domains) [30]. Domain scores were converted to 0–100 according to the WHO manual. Because of the split-body design, participants completed the questionnaire once per time point, reflecting the overall quality of life.

Adverse events were actively assessed during scheduled visits only and recorded immediately after each session (erythema, perifollicular edema, superficial carbonization) as present/absent. No event required medical intervention.

### Changes to the registered protocol

During trial conduct, protocol deviations were implemented to enhance feasibility and standardization.

The planned body image outcome was removed prior to participant recruitment. This outcome was considered unlikely to be sensitive in the present context because the axilla is a low-visibility body region and, within a split-body design, participants would have limited ability to perceive side-to-side differences. Therefore, body image was not included in the final outcome set.

Following the pilot phase, the protocol was amended to keep fluence constant across sessions. Although stepwise fluence escalation had been initially planned, a fixed fluence was adopted to improve standardization and comparability between IPL and DL. Accordingly, fluence was maintained at 25 J/cm² throughout the treatment course.

Also, following the pilot phase, pain during the procedure was collected verbally using an 11-point NRS instead of the prespecified 10-cm VAS. This change was made because protective eyewear required during treatment made visual marking impractical and would have disrupted the procedure.

Recruitment was discontinued before reaching the target sample size (planned *n* = 62; enrolled *n* = 48) due to feasibility constraints. No prespecified interim analysis or stopping rules were in place, and the decision to stop enrollment was not based on statistical significance testing. A post hoc assessment based on the observed effect size suggested > 80% power for the primary endpoint.

### Statistical methods

Normality was assessed using the Shapiro–Wilk test. Continuous variables were summarized as mean ± standard deviation when normally distributed and as median (interquartile range) otherwise. Categorical variables were described as absolute and relative frequencies (%).

To evaluate longitudinal changes in hair count and hair shaft thickness, linear mixed-effects models were applied, considering the intention-to-treat principle [31]. The models included treatment modality (IPL vs. DL), time (S0–S5), and their interaction as fixed effects, with participant included as a random effect to account for within-subject correlation inherent to the split-body design. A compound symmetry covariance structure was specified for repeated measures. Estimated marginal means were calculated for each group and time point, and pairwise comparisons were performed with Bonferroni adjustment for multiple testing.

Pain intensity (NRS, 0–10) was analyzed using Friedman tests for within-modality changes over time and Wilcoxon signed-rank tests for paired between-modality comparisons at each session (Bonferroni-adjusted). Binary adverse events were compared between modalities at each session using McNemar’s test and across sessions within each modality using Cochran’s Q, with Bonferroni-adjusted post hoc McNemar tests.

Per-protocol analyses were performed for hair outcomes, pain, and adverse events. At follow-up, between-modality comparisons for hair count and hair shaft thickness were performed using the Wilcoxon signed-rank test due to reduced sample size.

WHOQOL-bref domain scores (baseline vs. FU) and ordinal outcomes for satisfaction and modified GAIS were compared between modalities, when applicable, using the Wilcoxon signed-rank test. Statistical analyses were performed using IBM SPSS Statistics (version 30.0; IBM Corp., Armonk, NY, USA).

## Results

**Fig. 2:**
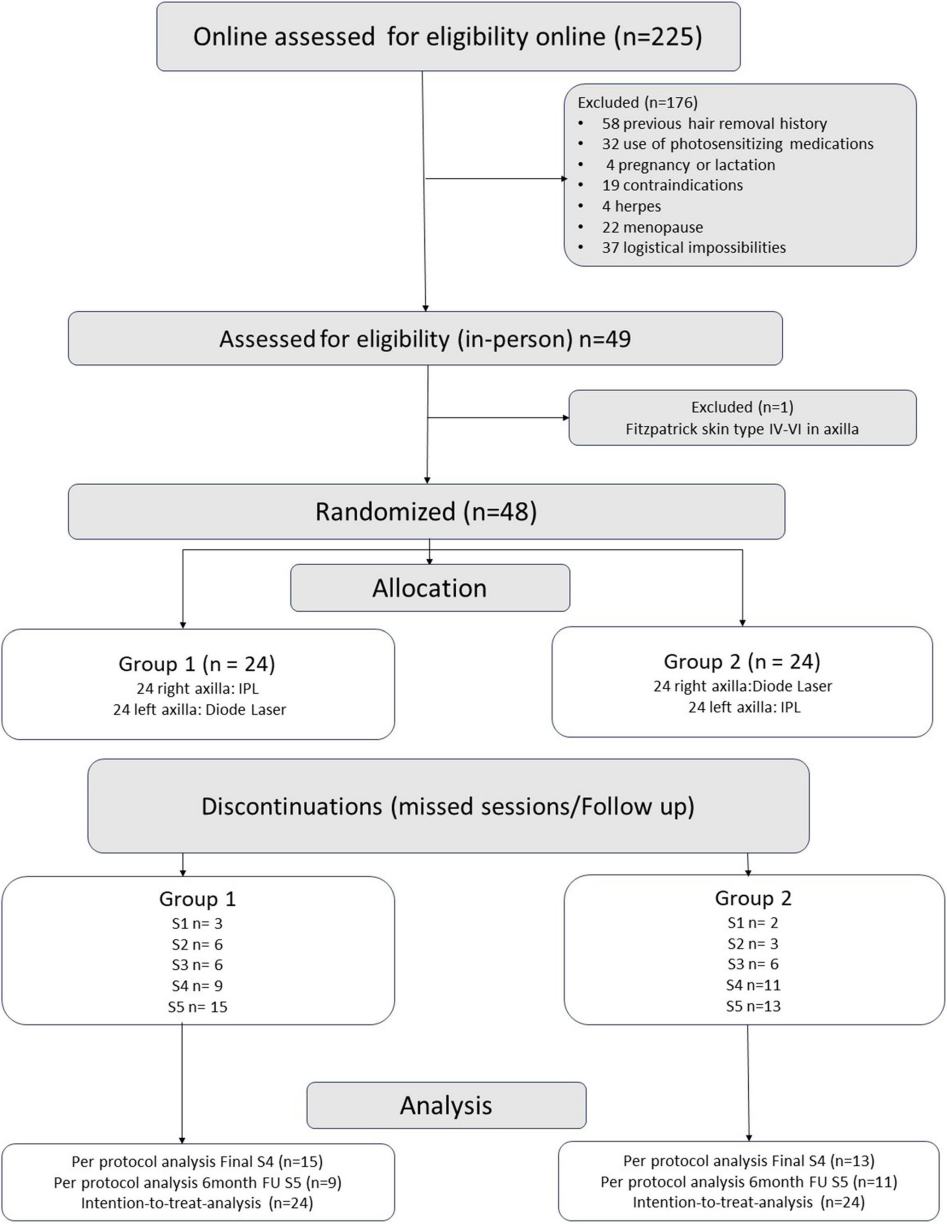
Research flowchart. The *n* value refers to the number of individuals (axilla is the n*2, being every patient treated with both IPL and DL)

A total of 225 women completed the enrollment form (Fig. 2). After screening, 176 were excluded due to specific exclusion criteria. Forty-nine participants underwent in-person assessment; one was excluded due to Fitzpatrick skin type VI in the treatment area, resulting in 48 women who initiated the protocol and provided written informed consent.

In this split-body trial, each participant received both modalities (IPL in one axilla and DL in the contralateral axilla), with randomized side allocation (24 assigned to IPL-right/DL-left and 24 to IPL-left/DL-right). All participants completed baseline assessment (S0) and received the first session. Attendance decreased over time, with missed visits increasing at each time point in both groups. At S4 (primary endpoint), 28 participants were assessed (9 missing on Group 1 and 11 missing in Group 2), and 20 completed the follow-up (FU) assessment (15 missing in Groups 1 and 13 missing in Groups 2). Both intention-to-treat and per-protocol analyses were performed for hair count and thickness.

Baseline data are presented in Table 2. Median age was 25.5 years, most participants were single (73%) and presented Fitzpatrick skin types III (42%) or IV (38%). Continuous or periodic medication use was reported by 42%, mainly oral or injectable contraceptives prescribed for contraception or management of polycystic ovary syndrome. Overall treatment exposure was high: the median number of sessions completed was 4, and 36 participants completed all four sessions (75%).

**Table 2:** Sample characterization (*n* = 48)

Variable	Value
Age (years)	25.5 [22–38.7]
**Marital status**	
Single	35 (73%)
Married	11 (23%)
Widow	2 (4%)
**Fitzpatrick skin type**	
I	1 (2%)
II	9 (19%)
III	20 (42%)
IV	18 (38%)
**Medication use**	20 (42%)
Number of sessions	4 [3.2–4.0]
**Attendance at sessions**	
First	48 (100%)
Second	43 (90%)
Third	39 (83%)
Fourth	36 (75%)
4-week assessment	28 (58%)
30-week follow-up	20 (42%)

* Median [25%–75%], absolute frequency (relative frequency %)

At baseline, hair counts per square centimeter were similar between modalities (*p* = 0.835) (Table 3). Hair counts decreased significantly over time in both modalities (time effect, *p* = 0.001), with a transient increase at S3 followed by further reductions at S4 and FU. For IPL, hair counts decreased from 11.1 ± 0.50 at baseline to 3.2 ± 0.63 at S4 (*p* < 0.001). For DL, counts decreased from 11.0 ± 0.50 to 1.6 ± 0.63 at S4 (*p* < 0.001). At S4 (primary endpoint), no statistically significant between-modality difference was observed (*p* = 0.074). At FU, hair counts were 3.2 ± 0.73 for IPL and 0.56 ± 0.73 for DL, with a significantly greater reduction in the DL group (*p* = 0.010). The mean between group hair count at FU was 2.6 hair/cm^2^, 95%CI 0.63 to 4.8.

**Table 3:** Follow-up of variables session by session, according to the intention-to-treat analysis

	Hair count (*n*/cm^2^)			Hair thickness (mm)		
	IPL *n* = 48	DL *n* = 48	Between groups *p*-value	IPL *n* = 48	DL *n* = 48	Between groups *p*-value
**Baseline**	11.1 ± 0.50^a^	11.0 ± 0.50^f^	0.835	0.183 ± 0.005^j^	0.178 ± 0.005^l^	0.501
**S1**	5.4 ± 052^b^	3.6 ± 0.52^g^	**0.016**	0.128 ± 0.006^j^	0.122 ± 0.006^m^	0.436
**S2**	3.4 ± 0.54^c^	2.8 ± 0.54^g^	0.440	0.087 ± 0.006^j, k^	0.089 ± 0.006^l, m,n^	0.765
**S3**	6.3 ± 0.56^b, d^	3.5 ± 0.56^g, h^	**0.001**	0.074 ± 0.006^j^	0.070 ± 0.006^n^	0.637
**S4**	3.2 ± 0.63^c, e^	1.6 ± 0.63^g^	0.074	0.048 ± 0.007^k^	0.043 ± 0.007^o^	0.571
**FU**	3.2 ± 0.73^b, c,e^	0.56 ± 0.73^g, i^	**0.010**	0.053 ± 0.008^k^	0.024 ± 0.008^p^	**0.010**
**Within group** **p-value**	**< 0.001**	**< 0.001**		**< 0.001**	**< 0.001**	

Mean ± Standard Deviation. S1 = session 1; S2 = session 2; S3 = session 3; S4 = session 4; FU = 30-week follow-up after the last session. Bold values highlight comparisons with p < 0.05; identical letters/symbols indicate p > 0.05 between time points

Baseline hair shaft thickness was similar between modalities (approximately 0.180 mm, *p* = 0.501). Thickness decreased significantly over time for both modalities (time effect, *p* < 0.001), with no significant between-modality differences up to S4 (Table 3). At FU, hair shaft thickness was 0.053 ± 0.008 mm for IPL and significantly lower for DL (0.024 ± 0.008 mm; *p* = 0.010). The mean between group hair thickness difference at FU was 0.029 mm 95%CI 0.007 to 0.051.

Given substantial missing data at S4 and FU, per-protocol analyses were performed (analysis sets differed by outcome - Table 4). Overall, per-protocol findings were consistent with the longitudinal mixed-model (intention-to-treat) analysis.

**Table 4:** Follow-up of variables session by session, according to the per protocol analysis

	Hair count (*n*/cm^2^)			Hair thickness (mm)			Pain intensity (0–10 NRS)				Adverse effects AE(%)				
	IPL *n* = 28	DL *n* = 28	Between groups *p*-value	IPL *n* = 28	DL *n* = 28	Between groups *p*-value	IPL *n* = 36		DL *n* = 36	Between groups *p*-value	IPL *n* = 36		DL *n* = 36		Between groups *p*-value
**Baseline**	10.5 [7.3–14.4]^a^	9.6 [8.5–15.0]^f^	0.863	0.188 [0.158–0.210]^j^	0.174 [0.148–0.215]^o^	0.357									
**S1**	3.6 [2.5–9.3]^b^	3.2 [2.4–4.9]^g^	0.222	0.138 [0.101–0.184]^k^	0.106 [0.102–0.128]^p^	0.568	1.0 [1.0–1.0]*	6.0 [5.0–7.0]^@^		**< 0.001**	ER ED C	34 (94.4%)^a^ 2 (5.6%)^f^ 1 (2.8%) ^i^	ER ED C	6 (16.7%)^e^ 30 (83.3%) ^g^ 25 (69.4%) ^j^	**< 0.001** **< 0.001** **< 0.001**
**S2**	3.4 [1.8–4.6]^c^	1.8 [0.94–3.9]^g, h^	0.214	0.090 [0.083–0.098]^l,n^	0.092 [0.083–0.094]^q^	0.111	1.0 [0.25–1.0]^#^	5.0 [5.0–6.0]^@^		**< 0.001**	ER ED C	23 (63.9%) ^b^ 5 (13.9%) ^f^ 0 (0%) ^i^	ER ED C	8 (22.2%) ^e^ 27 (75.0%) ^g, h^ 12 (33.3%) ^k^	**0.007** **< 0.001** **< 0.001**
**S3**	5.2 [2.7–8.4]^b, d^	3.8 [2.2–5.2]^g, h^	**0.001**	0.078 [0.070–0.092]^l.n^	0.070 [0.064–0.080]^r^	0.293	1.0 [0.25–1.0] *^,#^	5.0 [4.0–5.0]^&,$^		**< 0.001**	ER ED C	13 (36.1%) ^c^ 4 (11.1%) ^f^ 0 (0%) ^i^	ER ED C	11 (30.5%) ^e^ 21 (58.3%) ^h^ 5 (13.9%) ^l^	0.727 **< 0.001** 0.063
**S4**	2.6 [1.2–4.9]^c^	1.8 [0.94–2.4]^h^	**0.048**	0.051 [0.043–0.063]^m, n^	0.045 [0.034–0.062]^s^	0.413	0 [0–1.0]^§^	4.0 [4.0–5.0]^$^		**< 0.001**	ER ED C	9 (25.0%)c 4 (11.1%) ^f^ 0 (0%) ^i^	ER ED C	12 (33.3%) ^e^ 20 (55.6%) ^h^ 3 (8.3%) ^l^	0.648 **< 0.001** 0.250
**FU** *n* = 20	2.1 [1.2–3.3]^b, c,e^	0.5 [0–0.81]^i^	**< 0.001**	0.050 [0.041–0.062]^n^	0.032 [0.0–0.041]^t^	**< 0.001**									
**Within group** **p-value**	**0.001**	**0.001**		**0.001**	**0.001**		**0.001**	**0.001**			ER ED C	**< 0.001** 0.516 0.392	ER ED C	0.266 **0.011** ** < 0.001**	

Median [IQR], absolute frequency (%). Adverse effects: ED = edema; ER = erythema; C = carbonization. S1 = session 1; S2 = session 2; S3 = session 3; S4 = session 4; FU = 30-week follow-up after the last session. Bold values highlight comparisons with p < 0.05; identical letters/symbols indicate p > 0.05 between time points

For hair count, the mixed-model analysis did not detect a statistically significant between-modality difference at S4, whereas per-protocol analysis (*n* = 28) showed a significant difference favoring DL. At FU (*n* = 20), both approaches consistently demonstrated a greater reduction in hair count with DL, supporting the robustness of the observed long-term effect. For hair shaft thickness, between-modality differences at FU were also consistent across analyses, with no difference between modalities at S4 and with DL showing significantly lower values than IPL at FU.

Additionally, percentage reduction in hair count was also analyzed using per-protocol data. At the primary endpoint (S4), the DL-treated sites showed a greater median percentage reduction compared to IPL [DL: 87.5% (IQR 83.3–94.2) vs. IPL: 76.6% (IQR 65.0–89.9)], with a statistically significant difference between groups (Wilcoxon test, *p* = 0.001, *n* = 28). This difference remained at the final follow-up (S5), with DL continuing to demonstrate higher median reduction [DL: 96.0% (IQR 90.2–100) vs. IPL: 77.9% (IQR 65.1–88.6); *p* < 0.001, *n* = 20].

Pain perception decreased significantly over the sessions within each modality (Friedman test: IPL *p* = 0.001; DL *p* = 0.001). In IPL, a significant reduction was observed only between S1 and S4 (Bonferroni-adjusted *p* = 0.001). In DL, pain scores were similarly high at S1 and S2 (Bonferroni-adjusted *p* = 0.086) and similarly lower at S3 and S4 (Bonferroni-adjusted *p* = 0.999). Pain was consistently lower with IPL than with DL at each session (paired between-modality comparisons at each time point; Bonferroni-adjusted *p* = 0.001 for all between-modality comparisons). At S1, pain was higher with DL (median 6.0; IQR 5.0–7.0) than with IPL (median 1.0; IQR 1.0–1.0), and this between-modality difference persisted at S4 (DL: median 5.0, IQR 4.0–5.0; IPL: median 0, IQR 0–1.0).

Adverse events differed between modalities across sessions. Erythema decreased significantly over the sessions in IPL occurring in 94% and 62.5% of participants at S1 and S2, and decreasing to 27.1% (S3) and 29.2% (S4), in DL, erythema remained stable (~ 17–29%). Between-modality comparisons (McNemar) showed significant differences at S1 (*p* < 0.001) and S2 (*p* = 0.001), but not at S3 (*p* = 0.180) or S4 (*p* = 0.999). Perifollicular edema was consistently more frequent with DL (around 60%–83%) than with IPL (around 6%–17%) (McNemar: *p* < 0.001 at S1-S4).

Carbonization was also more frequent with DL across sessions (~ 15%–71%), than with IPL (0%–2%) (McNemar: S1 *p* < 0.001; S2 *p* < 0.001; S3 *p* = 0.004; S4 *p* = 0.016). Within DL, carbonization varied over sessions (Cochran’s Q *p* < 0.001); decreasing from S1 to S3 (71 to 19%), with no difference between S3 and S4 (*p* = 0.500); no meaningful variation occurred within IPL (Cochran’s Q *p* = 0.392). No adverse event required medical intervention, no serious adverse events occurred, and all events resolved within 30 days.

Descriptive stratification of adverse events by Fitzpatrick skin type (I–II, III, and IV) showed similar patterns across phototypes (Table S1, Supporting Information File). Erythema was more frequent in early sessions for both modalities, particularly with IPL, whereas perifollicular edema was more commonly observed with DL. Carbonization occurred mainly in intermediate sessions and showed no clear pattern across skin types. Overall, no consistent increase in adverse-event frequency was observed with higher phototypes.

Participant-reported outcomes and blinded photographic grading are presented in Table 5. Perceived discomfort during sessions was higher with DL than with IPL (median 4.0 [IQR 4.0–4.0] vs. 2.0 [IQR 2.0–2.0], *p* < 0.001), while discomfort duration was shorter in the DL group, being predominantly reported in two session (*p* = 0.004). Satisfaction with results favored DL (*p* = 0.005), and perceived improvement was higher with DL (median 4.0 [IQR 3.0–4.0]) than with IPL (median 3.0 [IQR 2.5–3.0]). The perceived improvement was higher for DL 4.0 (IQR 3.0–4.0) when compared to IPL 3.0 (IQR 2.5–3.0).

**Table 5:** Participant perception and professional evaluation

	IPL *n* = 20	DL *n* = 20	*p*-value
Discomfort ^a^	2.0 (2.0–2.0)	4.0 (4.0–4.0)	< 0.001
Duration of discomfort ^b^	2.0 (2.0–3.0)	2.0 (2.0–2.0)	**0.004**
Satisfaction with results ^c^	4.0 (4.0–4.0)	4.0 (4.0–5.0)	**0.005**
Perceived improvement (%) ^d^	3.0 (2.5–3.0)	4.0 (3.0–4.0)	**< 0.001**
GAIS (evaluator) ^e^	4.0 (3.0–4.0)	3.0 (3.0–3.0)	**0.011**

Discomfort, satisfaction, and participant-reported improvement according to self-assessment and GAIS (Global Aesthetic Improvement Scale, determined by evaluator), per protocol analysis. Values are presented as median (25%–75%)

^a^ Discomfort: 1 = very comfortable; 2 = comfortable; 3 = moderately comfortable; 4 = uncomfortable; 5 = very uncomfortable; ^b^Duration of discomfort: 0 = none; 1 = 1session; 2 = 2 sessions; 3 = 3 sessions; 4 = 4 sessions; ^c^ Satisfaction with results: 1= very dissatisfied; 2 = dissatisfied; 3 = slightly satisfied; 4 = satisfied; 5 = very satisfied; ^d^ Perceived improvement (%): 1 = up to 25%; 2 = up to 50%; 3 = up to 75%; 4 = up to 100%. GAIS: 0 = no results; 1 = poor; 2 = fair; 3 = good; 4 = excellent

**Fig. 3:**
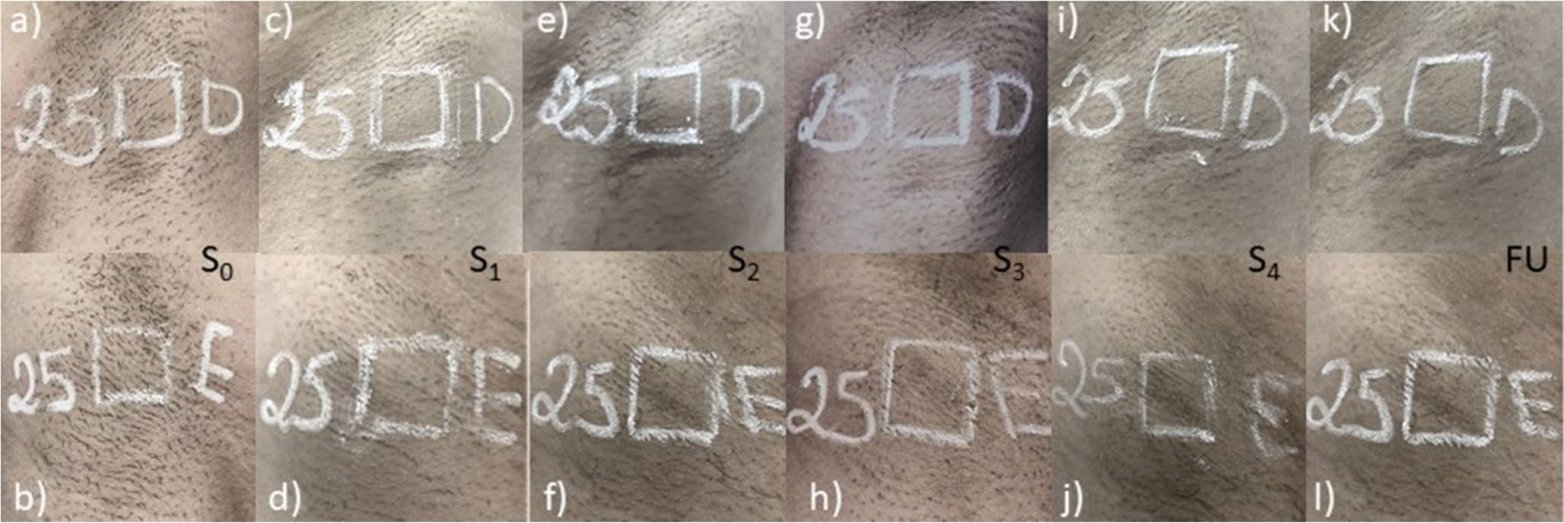
Macroscopic images of the treated regions. Longitudinal macroscopic images of the axillary regions from a representative participant skin type II assigned to IPL-right/DL-left. Images are presented side-by-side for each time point: baseline (S0), after sessions 1–4 (S1–S4), and follow-up (FU, 30 weeks after the final session). Upper row (**a**, **c**, **e**, **g**, **i**, **k**): right axilla treated with IPL. Lower row (**b**, **d**, **f**, **h**, **j**, **l**): left axilla treated with DL. The marked square indicates the standardized evaluation area used for hair count analysis. All images correspond to the same participant and anatomical regions, acquired under consistent conditions across time points; minor variations in illumination were adjusted for display purposes

Furthermore, the Global Aesthetic Improvement Scale (GAIS), assessed by a blinded evaluator, suggested excellent results for IPL (4.0 (IQR 3.0–4.0) while good results were found for DL (3.0 (IQR 3.0–3.0). Photographs were standardized according to the protocol shown in Fig. 3 to ensure uniformity of blinded assessment.

WHOQOL-bref results are presented in Table 6. Median scores in the Physical, Psychological, Social Relationships and Environmental domains remained stable throughout follow-up, and no statistically significant differences were observed between baseline and 30-week FU. The intervention did not exert a significant impact on the quality-of-life domains evaluated in this study.

**Table 6:** Quality of life determined by per protocol analysis (n is the number of individuals)

	Baseline	FU	*p*-value
WHOQOL Physical	78.6 (67.8–85.7)	78.5 (71.4–85.7)	0.731
WHOQOL Psychological	70.3 (62.5–83.3)	79.2 (66.7–83.3)	0.261
WHOQOL Social Relationships	75.0 (66.7–91.7)	75.0 (58.3–91.7)	0.704
WHOQOL Environment	62.5 (53.1–75.0)	65.2 (59.4–71.9)	0.549

Values are presented as median (25th–75th percentile)

## Discussion

In this split-body randomized trial, both IPL and DL significantly reduced axillary hair counts and hair shaft thickness over time. At the primary endpoint (S4), no statistically significant between-modality difference in hair count was observed in the mixed-model analysis, although complementary per-protocol analysis suggested a difference favoring DL. At follow-up, both analytical approaches consistently demonstrated superior long-term outcomes with DL. A similar pattern was observed for hair shaft thickness, with no significant between-modality differences up to S4 and lower values for DL at follow-up, supporting a sustained treatment effect over time. In contrast, IPL consistently showed lower pain scores throughout treatment, highlighting a clinically relevant trade-off between durability and tolerability.

The observed differences are plausibly explained by key technical distinctions between modalities. IPL, delivered through a 690-nm filter, provides noncoherent, polychromatic emission, whereas 810-nm diode laser delivers collimated, coherent light with high energy concentration at the target, which may favor more efficient follicular heating and selective photothermolysis [10, 20, 32, 33].

While previous comparative studies have reported a tendency toward greater long-term hair reduction with DL, findings remain heterogeneous and dependent on study design and treatment protocols. For example, Ormiga et al. [16] reported median reduction of 88.9% (IQR 73.9–92.3) with DL compared to 74.4% (IQR 46.2–84.2) with IPL at 6-month follow-up, using a protocol with progressive parameter escalation. In contrast, our study employed a larger intention-to-treat population, longitudinal repeated assessments, and standardized treatment parameters across devices. Under these controlled conditions, DL demonstrated greater sustained efficacy at follow-up (median reduction 96%, IQR 90.2–100) compared to IPL (77.9%, IQR 65.1–88.6), despite the use of fixed fluence and a reduced number of sessions. These findings suggest that treatment outcomes may be influenced not only by protocol design but also by intrinsic characteristics of the technologies.

Despite the use of cooled handpieces, discomfort remained higher with DL, consistent with the greater immediate follicular thermal effect observed with this modality. Cooling may reduce, but not eliminate, pain when thermal injury is more intense. While this pattern has been described in previous studies, including Ormiga et al. [16] direct comparisons are limited by methodological variability, particularly differences in fluence adjustment and treatment protocols. In our study, fluence and pulse duration were intentionally held constant across sessions to minimize confounding related to parameter escalation. Notably, significant hair reduction was achieved without increasing fluence, supporting the interpretation that differences in efficacy and tolerability are not solely protocol-dependent but reflect intrinsic characteristics of the technologies. Adjunctive strategies (e.g., topical anesthetics) may therefore improve tolerability in selected cases, particularly in high-density sites [34]. From a clinical perspective, these findings reinforce the importance of balancing efficacy and tolerability when selecting treatment parameters, rather than relying exclusively on progressive increases in energy delivery.

Adverse-event profiles differed between modalities. IPL was associated with higher erythema rates during early sessions, whereas DL more frequently induced perifollicular edema and carbonization, consistent with a stronger immediate follicular thermal effect and more concentrated energy delivery [35, 36]. These events were transient and required no medical intervention, supporting the overall safety of both modalities under the parameters used. Nonetheless, given that tolerability and short-term reactions affect adherence and perceived value, understanding these profiles may help guide individualized treatment selection [9]. Exploratory stratification by Fitzpatrick skin type did not demonstrate a consistent pattern of increased adverse events in higher phototypes, suggesting that both modalities were well tolerated across skin types I–IV under the evaluated conditions.

Quality of life scores (WHOQOL-bref) did not change significantly after treatment, consistent with previous reports [4]. However, this instrument is not specific to aesthetic interventions; dermatology-focused measures may be more sensitive to detect psychosocial changes related to hair removal. Future trials may benefit from incorporating condition- or dermatology-specific tools alongside generic measures.

Taken together, the objective outcomes indicate a durable advantage of DL at longer follow-up, whereas IPL demonstrates superior tolerability. This balance is clinically relevant, as pain and short-term adverse effects may influence treatment adherence and, consequently, real-world effectiveness. Importantly, these findings address key methodological limitations of previous studies, particularly heterogeneity in study design and the lack of standardized longitudinal assessment. By combining an intraindividual randomized design with standardized treatment parameters and repeated evaluations over time, this study provides a more controlled assessment of modality-specific effects compared to prior trials.

Key strengths of this study include the split-body design, objective outcome quantification, repeated evaluations across sessions, blinded outcome evaluation, and a relatively long FU period. Using each participant as her own control reduces interindividual variability and increases statistical efficiency. Although minor asymmetries between sides cannot be excluded, different body sites within the same individual tend to respond similarly to treatment [37]. Randomization of treatment side further minimizes systematic bias, and no carry-over effect is expected. However, this design may limit participants’ ability to perceive subtle differences between sides, potentially affecting patient-reported outcomes.

The longitudinal design also enabled the use of linear mixed-effects models for intention-to-treat analysis, incorporating all available data despite attrition and providing a more robust evaluation than complete-case approaches. In addition, standardization of treatment parameters across devices reduced confounding and allowed differences to be more directly attributed to intrinsic technological characteristics.

Our sample included a broad distribution of Fitzpatrick skin types (I–IV), with a predominance of types III and IV, enhancing the external validity for mixed-skin-tone populations. The 30-week follow-up was sufficient to assess durability, given the axillary hair cycle, and confirmed sustained superiority of DL despite a reduced number of sessions and fixed fluence [38].

Limitations include substantial loss to follow-up at S4 and FU, which may introduce attrition bias if missingness was not completely at random. However, mixed-model analyses were used to mitigate this limitation under the missing-at-random assumption.

Operator blinding was not feasible, and GAIS was assessed by a single evaluator, limiting reliability and precluding assessment of inter-rater variability. Satisfaction was measured using investigator-developed single-item measures rather than a validated instrument and should be interpreted cautiously. The divergence between participant satisfaction and evaluator-rated GAIS reflects differences in construct: satisfaction captures perceived benefit and durability, whereas GAIS represents standardized external assessment.

Hair count was measured using manual ImageJ analysis, which may introduce intra-observer variability and limited scalability. Nevertheless, this approach remains widely used as a practical reference method, particularly in the presence of image artifacts [39, 40]. Standardized acquisition and evaluation procedures were implemented to improve reliability. These limitations should be considered in interpreting the findings but do not undermine their overall consistency.

## Conclusion

In conclusion, both modalities were effective for axillary hair reduction. Diode laser demonstrated superior long-term objective outcomes, whereas IPL consistently showed better tolerability. These findings highlight a clinically relevant trade-off between efficacy and patient comfort, supporting individualized treatment selection based on patient expectations, pain tolerance, and skin type. Future studies with improved retention and standardized patient-reported outcomes are needed to further refine modality selection and optimize clinical outcomes.

## Supplementary Information

Below is the link to the electronic supplementary material.

Supplementary File 1 (DOCX 30.2 KB)

## Data Availability

A spreadsheet with tabulated and anonymized data will be available upon correspondence with the author. The spreadsheet is stored at the Mendeley data (https://data.mendeley.com/DOI:10.17632/8z9htg8tdd.1) and it may be accessed upon permission from authors. Pictures of the participants will not be made available at any time.
