# Supplementary material for: Efficacy and safety of intense pulsed light compared to diode Laser for hair removal: a randomized controlled trial
Source: Lasers Med Sci. 2026 Jun 6;41(1):108. doi: 10.1007/s10103-026-04904-6 (PMC13242373; doi:10.1007/s10103-026-04904-6)
Supplement: Supplementary file 1 — (DOCX 30.2 KB) [file 10103_2026_4904_MOESM1_ESM.docx]

| **Distribution of adverse events by modality and Fitzpatrick skin type across treatment sessions (n/N)** | | | | | | | | | |
| --- | --- | --- | --- | --- | --- | --- | --- | --- | --- |
|  | **IPL  (n/N within skin type)** | | | | **DL  (n/N within skin type)** | | | |  |
|  |  | **ST I-II** | **ST III** | **ST IV** |  | **ST I-II** | **ST III** | **ST IV** |  |
| S1  N=48 | ER  ED  C | 10/10  0/10  0/10 | 18/20  2/20  1/20 | 17/18.  1/18  1/18 | ER  ED  C | 6/10  4/10  1/10 | 15/20  5/20  5/20 | 12/18  6/18  1/18 |  |
|  |  |  |  |  |  |  |  |  |  |
| S2  N=43 | ER  ED  C | 5/8  2/8  8/8 | 10/19  2/19  18/19 | 8/16  5/16  17/16 | ER  ED  C | 5 / 8  3 / 8  8 / 8 | 9/19  3/19  18/19 | 12/16  4/16  17/16 |  |
|  |  |  |  |  |  |  |  |  |  |
| S3  N=41 | ER  ED  C | 4/8  0/8  0/8 | 5/18  2/18  0/18 | 5/13  3/13  0/13 | ER  ED  C | 3 / 8  1 / 8  0 / 8 | 5/18  1/18  0/18 | 7/13  3/13  0/13 |  |
|  |  |  |  |  |  |  |  |  |  |
| S4  N=36 | ER  ED  C | 4/7  0/7  0/7 | 5/16  1/16  0/16 | 3/13  2/13  0/13 | ER  ED  C | 1 / 7  2 / 7  0 / 7 | 3/16  1/16  0/16 | 7/13  4/13  0/13 |  |

Table S1: Descriptive distribution of adverse events by modality and Fitzpatrick skin type across treatment sessions (n/N within skin type). Adverse effects: ED = edema; ER = erythema; C = carbonization. S1 = session 1; S2 = session 2; S3 = session 3; S4 = session 4.
